# Drying and Rainfall Shape the Structure and Functioning of Nitrifying Microbial Communities in Riverbed Sediments

**DOI:** 10.3389/fmicb.2018.02794

**Published:** 2018-11-16

**Authors:** Maria Isabel Arce, Daniel von Schiller, Mia M. Bengtsson, Christian Hinze, Hoseung Jung, Ricardo J. Eloy Alves, Tim Urich, Gabriel Singer

**Affiliations:** ^1^Leibniz-Institute of Freshwater Ecology and Inland Fisheries, Berlin, Germany; ^2^Department of Plant Biology and Ecology, University of the Basque Country (UPV/EHU), Bilbao, Spain; ^3^Institute of Microbiology, University of Greifswald, Greifswald, Germany; ^4^Integrative Research Institute on Transformations of Human-Environment Systems (IRI THESys), Humboldt University of Berlin, Berlin, Germany; ^5^Department of Ecogenomics and Systems Biology, University of Vienna, Vienna, Austria

**Keywords:** intermittent, stream, nitrification, ammonia oxidation, AOA, AOB, NOB, COMAMMOX

## Abstract

Non-flow periods in fluvial ecosystems are a global phenomenon. Streambed drying and rewetting by sporadic rainfalls could drive considerable changes in the microbial communities that govern stream nitrogen (N) availability at different temporal and spatial scales. We performed a microcosm-based experiment to investigate how dry period duration (DPD) (0, 3, 6, and 9 weeks) and magnitude of sporadic rewetting by rainfall (0, 4, and 21 mm applied at end of dry period) affected stocks of N in riverbed sediments, ammonia-oxidizing bacteria (AOB) and archaea (AOA) and rates of ammonia oxidation (AO), and emissions of nitrous oxide (N_2_O) to the atmosphere. While ammonium (NH_4_^+^) pool size decreased, nitrate (NO_3_^−^) pool size increased in sediments with progressive drying. Concomitantly, the relative and absolute abundance of AOB and, especially, AOA (assessed by 16S rRNA gene sequencing and quantitative PCR of ammonia monooxygenase genes) increased, despite an apparent decrease of AO rates with drying. An increase of N_2_O emissions occurred at early drying before substantially dropping until the end of the experiment. Strong rainfall of 21 mm increased AO rates and NH_4_^+^ in sediments, whereas modest rainfall of 4 mm triggered a notable increase of N_2_O fluxes. Interestingly, such responses were detected only after 6 and 9 weeks of drying. Our results demonstrate that progressive drying drives considerable changes in in-stream N cycling and the associated nitrifying microbial communities, and that sporadic rainfall can modulate these effects. Our findings are particularly relevant for N processing and transport in rivers with alternating dry and wet phases – a hydrological scenario expected to become more important in the future.

## Introduction

Through channel-routing and aquatic processes, fluvial networks have the capacity to modify global pools of nitrogen (N). Unless evading to the atmosphere as dinitrogen gas (N_2_), nitrous monoxide (NO) or nitrous oxide (N_2_O) (Beaulieu et al., 2011), N that enters rivers mostly in headwaters (Peterson et al., 2001) is delivered to downstream and coastal ecosystems in organic or remineralized form (Hall et al., 2009; Howarth et al., 2011). Part of N processing in fluvial networks is associated to rivers that cease to flow and become dry at some point in space and time (Datry et al., 2014; von Schiller et al., 2017). These intermittent rivers are a natural phenomenon at the global scale, with more than 50% of the global fluvial network estimated to be intermittent (Datry et al., 2014). This proportion is expected to rise in the face of increasing water demand and global warming (Palmer et al., 2008; Hoerling et al., 2012).

Dry riverbeds have historically been considered biogeochemically inert in comparison with running waters, but recent research identifies these habitats as considerable areas of nutrient transformation (e.g., Steward et al., 2012; Gómez-Gener et al., 2015). This is a critical aspect, because how N is processed during the dry phase strongly influences ecosystem functioning and N export upon flow recovery. Evidently, understanding dry-phase processing of N can help us better understand the role of intermittent rivers in N budgets at larger spatial scales.

Sediment drying exerts a strong impact on sediment biogeochemical processes and resultant pools of N in intermittent rivers (Austin and Strauss, 2011; Gómez et al., 2012; Arce et al., 2014). Overall, a significant number of microbial cells are destroyed during drying due to osmotic shock (van Gestel et al., 1991), affecting microbial communities and the biogeochemical pathways they control (Placella and Firestone, 2013; Pohlon et al., 2013). Yet, recent research indicates that nutrient processing in intermittent rivers can be maintained during dry periods to a certain extent by the activity of microbial communities adapted to low moisture conditions (Zoppini and Marxsen, 2010; Timoner et al., 2012; Pohlon et al., 2013).

Based on redox requirements, changes in the relative importance of N cycling pathways are expected to occur during the transition from wet to dry conditions. As sediment becomes exposed to the atmosphere during drying, the expanding oxic conditions reduce anaerobic processes such as denitrification (Austin and Strauss, 2011; Gómez et al., 2012) and favor aerobic pathways such as nitrification (Gómez et al., 2017).

Nitrification is a crucial process in N cycling bridging reduced with oxidized forms of dissolved inorganic N (Merbt et al., 2015). In the first step of nitrification, i.e., AO, ammonium (NH_4_^+^) is first oxidized to nitrite (NO_2_^−^) by two different groups of microorganisms, ammonia- oxidizing archaea (AOA) and bacteria (AOB) and it is considered the rate-limiting step of nitrification crucial for the subsequent oxidation to nitrate (NO_3_^−^) which is mediated by NOB. Recently, the complete NH_4_^+^ oxidation to NO_3_^−^, termed COMAMMOX, has been identified in two species of the NOB genus *Nitrospira* (Daims et al., 2015; van Kessel et al., 2015).

Two studies (Gómez et al., 2012; Merbt et al., 2016) found higher AO rates and NO_3_^−^ concentrations in dry riverbed habitats compared to wet sediments suggesting that oxygenation associated to loss of water promotes this process. Such NO_3_^−^ enrichment tends to be more pronounced in surface sediments from where built-up NO_3_^−^ is prone to be easily flushed upon flow resumption. The observed build-up of NO_3_^−^ in dry riverbeds repeatedly led to the suggestion that dry period duration (DPD) may have implications for NO_3_^−^ export upon flow recovery in intermittent rivers (Gómez et al., 2012; Arce et al., 2014; Merbt et al., 2016). Few studies have combined approaches at the levels of microbial cell identities and ecosystem processes in intermittent rivers, so that links between the abundance of nitrifying microbes and N-processing rates are still unclear, especially in response to temporary water stress (Prosser, 2012). Some research results were even conflicting. For instance, although Merbt et al. (2016) found the higher AO rates in dry riverbeds, they detected no major changes in the abundance of *amoA* genes (encoding *amo*A) of both AOA and AOB across the different habitats (i.e., running waters, isolated pools and dry streambeds) of a drying Mediterranean stream. Archaea and bacteria have distinct cell structure and physiology, including osmo-adaptation strategies (Zillig, 1991; Roeßler and Müller, 2001); thus, differential functional responses to stream drying could be expected.

The no-flow period in an intermittent river does not imply a completely dry riverbed where biogeochemical processes pause and life is absent. In soils and river sediments during periods of water limitation, rewetting due to rainfall can trigger rapid biogeochemical responses (Austin et al., 2004; Schwinning et al., 2004; Collins et al., 2008) such as AO (Arce et al., 2014), N mineralization (McIntyre et al., 2009) and trace gas evasion such as N_2_O (Gallo et al., 2014). Such rain events, albeit sporadic, have the potential to buffer the impacts of drying on N transformation rates by temporarily stimulating bacterial growth rates (Lovieno and Bååth, 2008). For example, Placella and Firestone (2013) detected rapid transcriptional response of AOA, AOB, and NOB after months of desiccation-induced inactivation. Upon rewetting, release of intracellular osmolytes even from live cells can take place (Halverson et al., 2000; Schimel et al., 2007) and contribute to increase nutrient availability enabling “hot moments” of intense biogeochemical cycling (Borken and Matzner, 2009). Any internal increase in nutrients accompanied to vertical mobilization in response to rainfall may be especially crucial to feed microbial activity in deep layers where direct nutrient inputs (e.g., atmospheric N deposition) can be limited.

Furthermore, studies in soils have demonstrated that the magnitude of a water pulse (i.e., more or less water amount) controls microbial activity in the short-term (Fierer and Schimel, 2002; Shi and Marschner, 2014; Yu et al., 2014).

Combined molecular and biogeochemical information for the dynamics of AO during progressive drying in intermittent rivers is still scarce and no evidence exists about how the duration of the preceding dry period controls the nitrifying response to a water pulse. Understanding the responses of microbial community structure and functioning associated with changes in sediment moisture will contribute to increase our knowledge about the N cycle of intermittent rivers during non-flow periods, which are the less studied phases of these ecosystems (von Schiller et al., 2017).

We performed a microcosm-based experiment to assess (i) how progressive drying affects the abundance of nitrifying microbes and their functioning as well as N fluxes (dissolved and gaseous) across the sediment vertical profile and to the atmosphere, (ii) how rainfall events of various magnitude can affect such features, and (iii) whether any effects of rainfall depend on the duration of the preceding dry period. To this end, we combined molecular and biogeochemical approaches to study nitrification. We measured the relative abundance (RA) of AO functional groups through 16S rRNA gene sequencing and quantified *amoA* genes for AOA and AOB by quantitative PCR (qPCR). Further, we measured rates of AO. We predicted that the abundance and activity of AO microbes and consequently the sediment NO_3_^−^ concentration would increase from wet to dry conditions, and that this increase would be more pronounced in the more air-exposed surface sediments. We further predicted that rainfall would exert changes on AO rates and N fluxes with a magnitude proportional to the size of the water pulse.

## Materials and Methods

### Sediment Collection and Microcosm Set Up

We collected sediments from a 100-m reach of the lowland intermittent river Fredersdorfer Mühlenfließ (Brandenburg, Germany, 52°29′09.7″N, 13°42′39.4″E) in June 2015 before surface water disappearance. The occurrence of hydrological droughts in this river during 1–2 summer months has been documented since the 1990’s (Mey, 2003) and is mainly controlled by reductions of groundwater levels (Nützmann and Mey, 2007). Surface river water concentrations (mean ± SD, *n* = 3 replicates across the stream reach) at the time of sediment collection were 230 ± 25 μg N L^−1^ for NO_3_^−^, 360 ± 50 μg N L^−1^ for NH_4_^+^, 31 ± 2 μg P L^−1^ for soluble reactive phosphorus (orthophosphate, PO_4_^3−^), 10.6 ± 1.5 mg C L^−1^ for dissolved organic carbon (DOC) and 0.53 ± 0.21 mg N L^−1^ for dissolved organic nitrogen (DON; see next section for the different methods used). The collected sediments were mainly composed of sand (90–96% larger than 63 μm, according to *Ad hoc*-AG Boden 2005) and had an organic matter content (based on loss on ignition) of 8 ± 2%.

Submerged sediments were collected in three separate sampling points of the chosen stream reach at 0–15 cm depth. Sediments from each point were sieved (mesh size 4 mm), homogenized, individually kept in plastic tanks and transported to the lab in dark and cold conditions. Unfiltered surface stream water was collected in parallel. In the lab, 30 transparent acrylic glass columns (length 30 cm, diameter 5.5 cm) with gas-tight septa caps were filled with 15 cm of sediment. Surface stream water was added to all cores to completely wet them with a 2–3 cm water column. The filled microcosms were placed in a dark climate chamber at a constant temperature of 25°C. After acclimation to laboratory conditions for 1 week, three replicate microcosms were destructively analyzed for surface water and sediment characteristics representing the initial wet conditions (*t* = 0 weeks). Such initial conditions were thought to simulate a pool environment, the hydrological habitat that typically appears during the fragmentation phase following flow cessation and before complete surface drying (Steward et al., 2012). At the same time, overlaying water in the remaining 27 microcosms was drained through the sediment via silicone tubes at the bottom, and microcosms were then left to dry in the climate chamber for up to 9 weeks. Microcosms were grouped into 3 sets of 9 in a way that all of sediments from the three sampling sites were represented in each set. Each set was dedicated to one of three sampling occasions (3, 6, and 9 weeks of drying). Over the entire experiment (i.e., 9 weeks), the columns were weighed every 2 days to register changes in water content (WC) (Supplementary Figure S1).

Dissolved oxygen (DO) in the interstitial part of the sediment was monitored at two sediment depths (1.5 and 8 cm) over the entire experiment using non-invasive optode technology (Microx 4, PreSens, Regensburg, Germany). For this, DO sensors (circular, diameter 0.5 cm) were attached to the inner walls of the set of microcosms dedicated to the 9-week dry period. These measurements thus represented DO dynamics during the entire experiment without any rewetting (Supplementary Figure S1).

As a second experimental approach besides DPD, we used artificial rainwater to generate water pulses of two different magnitudes and after ending each dry period (3, 6, and 9 weeks). We selected rainfalls magnitudes (RM) of 4 mm (modest rainfall) and 21 mm (intense rainfall) based on historical monthly data of precipitation during the summer season in the region of the stream used for sediment collection. Water was applied by spraying it on sediments. A third treatment of 0 mm (no rainfall) served as a control (Supplementary Figure S1). This resulted in a two-factorial experimental design: DPD × RM with *n* = 3 for each design cell. We used available information of the chemical composition of rainfall in Northeast Germany (CarboZALF meteorological station). Briefly, chemical composition averaged 1.4 mg L^−1^ of Cl^−^, 2.2 mg L^−1^ of SO_4_^2−^, 0.4 mg L^−1^ of K^+^, and 1.6 mg L^−1^ of Ca^2+^. Although natural rain contains inorganic N, the artificial rainwater was not amended with N to avoid potential interferences with N available in the sediment. In no case, rainfall events generated an overlaying water column.

### Water Sampling and Analysis

At initial conditions (*t* = 0 weeks), surface water samples from the surface of microcosms were collected with plastic syringes and filtered through combusted (4 h, 450°C) Whatman GF/F filters (Maidstone, England, United Kingdom; 0.7 μm pore size) mounted in syringe filter holders. Water was analyzed for NO_3_^−^ and NH_4_^+^ following the cadmium reduction (Wood et al., 1967) and Salicylate (Reardon et al., 1966) method using a segmented flow analyser (Skalar San^++^, Breda, Netherlands), for PO_4_^3−^ following the molybdate method (Murphy and Riley, 1962) on a Shimadzu (Kyoto, Japan) UV-1800 spectrophotometer, and for DON using size exclusion liquid chromatography equipped with an organic nitrogen detection system (LC-OCD-OND, DOC-Labor Huber, Karlsruhe, Germany). Concentrations of NO_3_^−^, NH_4_^+^ and DON in surface water from the wet microcosms (*t* = 0 weeks) were (mean ± SD, *n* = 3) 0.43 ± 0.16, 1.64 ± 0.20 and 0.77 ± 0.14 mg N L^−1^, respectively. The PO_4_^3−^ concentration was 36 ± 2 μg P L^−1^.

### Sediment Sampling and Analysis

During initial wet conditions (*t* = 0 weeks) and after *t* = 3, 6, and 9 weeks, sediments were destructively removed from a microcosm subset. After 3, 6, and 9 weeks, sediment sampling was done 24 h after rainfall treatments were applied. The sediments in surface (0–3 cm) and deep (3–15 cm) layers were separately collected and homogenized in plastic bags. Previous trials with the used microcosms in the climate chamber served us to decide these two layers are different enough on the base of drying and oxygenation over the 9 weeks of the total experimental time. Immediately after collection, subsamples (0.5 mL) for DNA extraction were stored in sterile plastic vials under liquid N and subsequently at −80°C. The remaining sediment samples were analyzed after collection for WC, concentration of N species and AO rates.

WC was determined after drying sediment sub-samples at 60°C for 72 h and expressed as % water (g/g). The concentrations of NO_3_^−^, NH_4_^+^ and DON in the sediments were measured after extraction with 2M KCl 1:5 (175 rpm, 25°C, 1 h). The resulting filtrates were analyzed as described above for water samples. Sediment contents were expressed as μg or mg of N per g of dry sediment (DM) after correcting by the WC% in the sediment.

We measured the AO rate as a proxy of nitrifying activity following the acetylene (C_2_H_2_) gas inhibition method at a partial pressure of 100 Pa (Offre et al., 2009). This method allows measurements in sediments under actual humidity, which is not possible when using other methods that involve the addition of the inhibitor in a water solution (Merbt et al., 2016). For each sediment replicate, two (125 mL) flasks fitted with septa caps were incubated with 10 g of sediments for 72 h, with or without C_2_H_2_ (inhibited/control flasks). Every 24 h, all bottles were opened for 30 min to ensure continuous aerobic conditions for nitrification and C_2_H_2_ was added again to the blocked flasks. At the end of the incubation, sediment NH_4_^+^ concentration was analyzed after conducting a KCl extraction as previously described. The quantity of NH_4_^+^ oxidized was calculated as the difference in extractable NH_4_^+^ between the inhibited and control flasks after incubation and was expressed as μg NH_4_-N g^−1^ DM h^−1^ (Levi et al., 2013).

### DNA Extraction, 16S rRNA Gene Sequencing and Analysis of Nitrifying Functional Groups

Total community DNA was extracted from 0.25-g sediment samples using the PowerSoil DNA isolation kit (MoBio Laboratories, CA, United States) according to the manufacturer’s instructions with exception of a bead beating step performed in a MP FastPrep-24 5G High Speed Homogenizer (MP Biomedical, CA, United States) during 30 s at a speed of 5 m/s. The extracted DNA was quantified using a NanoDrop spectrophotometer (measured concentrations ranged between 20 and 63 ng μl^−1^), expressed in ng per g of DM and used as a proxy of microbial biomass. DNA was amplified with primer pairs targeting the V4 region of the 16S ribosomal RNA (rRNA) gene for archaea and bacteria (515F and 806R; Walters et al., 2016). PCR amplification, Illumina MiSeq library preparation and paired-end sequencing (V3 chemistry) was carried out by LGC Genomics (Berlin, Germany). Sequence reads (clipped from adaptor and primer sequence remains) were processed using the DADA2 package in R (version 1.2.0) (Callahan et al., 2016; R Core Team, 2017). Chimeric sequences were removed using the removeBimeraDenovo function. The resulting amplicon sequence variants (ASVs, analogous to operational taxonomic units) were used to construct a table containing RAs of ASVs across all samples. ASVs were taxonomically classified with BlastN using a lowest common ancestor approach (Lanzén et al., 2012) on a manually curated version (silvamod) of the Silva database (Pruesse et al., 2007, SSURef version 123) with MEGAN parameters (top percent 2, minimum bit score 155, minimum number of hits 1). Nitrifier ASVs were identified and classified via a 2-step approach. First, AOA and AOB were identified among the Silva-classified ASVs based on the taxonomical affiliation of the 16S rRNA genes to known nitrifying genera, families and orders in the Silva database. Second, we further critically verified the affiliation to nitrifier groups of identified ASVs from step 1 by Blast against the Genbank 16S rRNA and nr databases. Despite not being the focus of our experiment, we tentatively identified COMAMMOX and NOB groups and provided data about their RA. Especially for AOB and NOB the classification with Silva resulted in too broad groups with not all identified sequences being from characterized AOB or NOB, respectively. For the verification of these potential nitrifier ASVs in step 2 we used different thresholds of sequence identity to reference sequences. Threshold for acceptance of ASVs as AOB were 93% sequence identity to known nitrifying species of the *Nitrosomonadaceae* (Betaproteobacteria) family and *Nitrosococcus* genus (Gammaproteobacteria) in the Genbank 16S rRNA database. For species and candidatus species of the AOA the threshold was 92% sequence identity and for NOB (*Nitrospirae*) the sequence identity threshold was 90%. The differing identity thresholds reflect the phylogenetic breadth of the target groups, i.e., family (*Nitrosomonadaceae*), order (*Nitrososphaerales* and *Nitrosopumilales*) and class (*Nitrospirae*). ASVs related to COMAMMOX were identified by BlastN against the 16S rRNA gene sequences of *Nitrospira inopinata* and *Nitrospira nitrosa* based on at least 98% sequence identity. Results were expressed as RAs of each group with respect to the whole community based on 16S rRNA. Illumina MiSeq 16S rRNA amplicon sequence data was submitted to the NCBI Short Read Archive under the accession number SRP137655.

Quantitative PCR of AOB *amoA* genes was performed with primer pair amoA-1F/amoA-2R (Rotthauwe et al., 1997). AOA *amoA* genes were amplified with primers CamoA-19F (5′-ATGGTCTGGYTWAGACG-3′; Tourna et al., 2008; Pester et al., 2012) and TamoA-629R (5′-GCCATCCATCKRTANGTCCA-3′; designed in this study), respectively. All qPCR amplifications were performed with two DNA template concentrations (2 and 5 ng μl^−1^) in duplicate 15 μL reactions each on an Analytik Jena qTower 2.2 cycler, as follows: 10 μL 2× innuMIX qPCR Master Mix (Analytik Jena), 10 μM of each primer for archaeal and bacterial *amoA*, respectively. Cycling conditions were as follows: 2 min initial denaturing step at 95°C, followed by 35 cycles of 30 s denaturing at 95°C, 30 s joint annealing-extension at 55°C for archaea and for bacteria, 45 s elongation at 72°C and 5 min final extension at 72°C, for archaea or bacteria, respectively. A plate read was included at the end of each cycle for 10 s at 72°C for archaea and for bacteria. Quantification of archaeal and bacterial *amoA* genes was based on serial dilutions (10–10^7^ gene copies) of M13-PCR products containing the *amoA* gene of *Nitrososphaera viennensis* or *Nitrosospira multiformis* ATCC25196, respectively. qPCR efficiencies for archaeal and bacterial *amoA* gene assays were 90–97% and 91–92%, respectively. Trend lines of duplicate standards from all assays had slope and Y-intercept values ranging from −3.39 to −3.6 and 31.35 to 39.6, respectively, all with *R*^2^ values ≥ 0.99, and with sensitivity down to 10 copies per reaction. Specific amplification was confirmed by melting curve analysis and standard 1% agarose gel electrophoresis after qPCR. Results were expressed as number of gene copies per g DM.

### N_2_O Fluxes Sampling and Analysis

Fluxes of N_2_O from sediments to atmosphere were measured at shorter intervals during the entire drying period, and during the rainfall events. Using the 9 microcosms set up for the longest DPD (i.e., 9-week) (Supplementary Figure S1), N_2_O dynamics were monitored at multiple times: before microcosm drainage (*t* = −3 days), immediately after drainage (*t* = 0 days) and several days after drainage and further desiccation (*t* = 3, 6, 9, 14, 20, 30, 42, 54, and 65 days). On each sampling day, these microcosms were capped gas-tight, and gasses were sampled from the headspace of the columns several times over 5 h. In addition, in the different sets of microcosms for the different DPDs, gas sampling was conducted during the simulated rainfall events over 5 monitoring phases: before (*t* = −2 h), immediately after (*t* = 0 h) and further 3 times after the water pulse (*t* = 2, 6, and 24 h). At the end of each monitoring phase, the caps of the microcosms were removed and the air inside was allowed to equilibrate with the atmosphere. The collected 20-mL gas samples were stored in 10-mL pre-evacuated glass vials with inert butyl rubber crimp caps (Machery-Nagel GmbH & Co., Berlin, Germany). N_2_O was analyzed on a gas chromatograph fitted with an electron capture detector (ECD; Shimadzu, Tokyo, Japan).

Gas fluxes were calculated via linear regression of gas concentrations versus time elapsed since the microcosm cap placement. We used microcosm headspace volume to compute the mass of N_2_O as μg of N at each sampling time and divided by the sediment area. We also considered the WC in sediments for correcting concentrations of gasses dissolved in water (Tiedje, 1982). Gas fluxes were expressed as μg N_2_O-N m^−2^ h^−1^. Finally, the cumulative fluxes over 24 h following the rainfall event (μg N_2_O-N m^−2^) were calculated by integrating the fluxes over time from the water pulse to the end of the 24-h period.

### Statistical Analysis

We addressed the effect of the DPD on the studied variables by comparing values under 0 mm (i.e., dry sediments) across the different times (*t* = 3, 6, and 9 weeks) with those measured under initial, wet conditions (i.e., *t* = 0 weeks) using Student’s *t*-test assuming unequal variances. We followed Holm’s method to adjust *P*-values in order to avoid type II error inflation due to excessive *P*-value adjustment. To examine the influence of RM (0, 4, and 21 mm) and whether it was controlled by the DPD (*t* = 3, 6, and 9 weeks), we then used mixed effects models that included RM and DPD as fixed factors and their interaction. Variance heterogeneity was common, and we thus used a flexible variance structure (Zuur et al., 2009). Mixed effects models were run in parallel for the two sediment layers to ease interpretation. When the interaction term RM x DPD was significant, specific differences among rainfall treatments fixed by time were also evaluated through Holm’s *post hoc* test. In the case of cumulative N_2_O fluxes, when significant RM effects were found, we explored differences in the fluxes before, immediately after and following the rainfall using similar mixed effect models. In this case, we included time (i.e., monitoring times = −**2**, 0, 2, 6, and 24 h), RM (0, 4 and 21 mm) and the interaction time × RM as factors within the model. Mixed models were used to adequately account for repeated measurements. After *P*-value adjustments results were considered significant at *P* ≤ 0.05. When necessary, simple linear regressions were used to assess the relationship between some variables and *R*^2^ and *P*-values were provided. A non-metric multidimensional scaling (nMDS) was also made using all OTUs as input data (all prokaryotes) to visualize potential linkages of AOA and AOB with certain chemical and biogeochemical variables. All the statistical analyses were performed using packages ‘nlme’ and ‘lsmeans’ in R version 3.2.2^1^. Multivariate analyses on microbial community data were carried out with functions of ‘vegan’ (Oksanen et al., 2016).

## Results

### Sediment WC and DO

**FIGURE 1:**
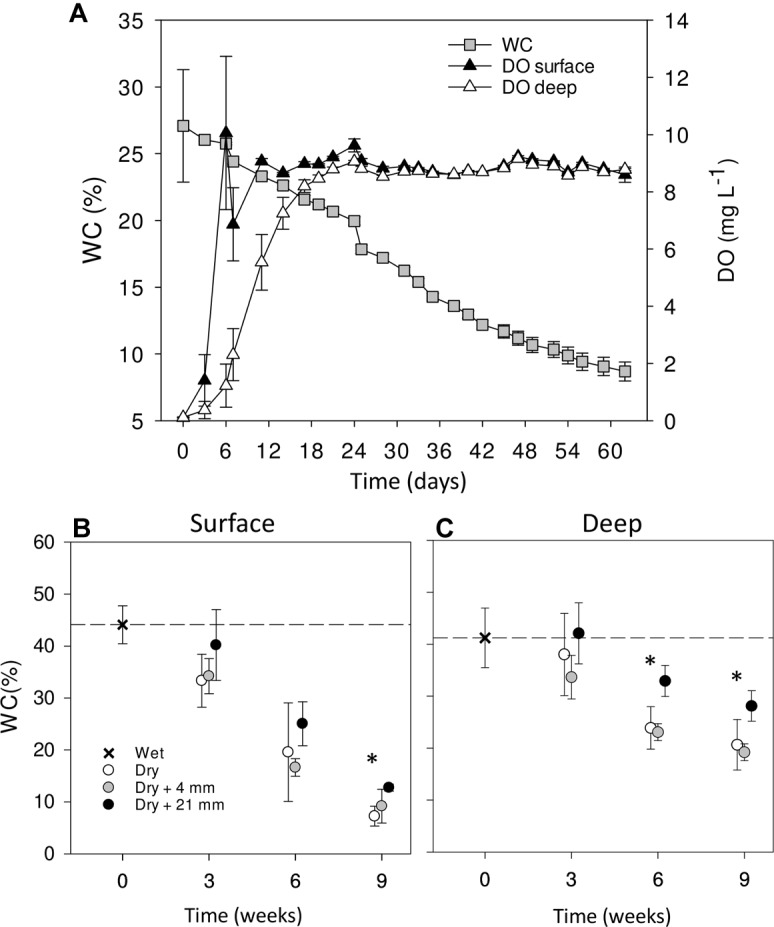
Mean (±SE, *n* = 9) WC and DO concentration in surface and deep sediments monitored over the entire experiment in dry microcosms with no rainfall application **(A)**. Mean WC (±SE, *n* = 3) in surface **(B)** and deep **(C)** sediments during initial conditions (0 weeks) and after the different RMs for each DPD. Asterisks (^∗^) denote significant (*P* ≤ 0.05) differences to initial wet conditions after Student’s *t*-test followed by Holm’s correction for multiple comparisons. The dashed line represents the initial conditions as a reference for a better comparison.

Over the course of the 9-week-experiment and without any simulated rainfall, the WC in the microcosms gradually decreased from ∼30% at initial wet conditions to ∼10% at the end of the experiment (Figure 1A). In parallel, DO in the sediments increased with desiccation converging to saturation at ∼9 mg L^−1^ after 20 days and then remaining stable throughout the rest of the experiment (Figure 1A). The surface layer dried faster (Figures 1B,C) and showed a faster increase in DO (Figure 1A) than the deep layer. When comparing WC% in the dry microcosms (i.e., 0 mm selected) with wet conditions (*t* = 0 weeks), we found significant differences only after 9 weeks in surface sediments (Figure 1B) and after 6 and 9 weeks in deep sediments (Figure 1C) (*t*-test, *P* < 0.001). In both cases, there were significant differences between RM regardless of the DPD (Table 1), with WC% generally higher under 21 mm rainfall.

**Table 1:** Statistical results from the mixed effects models of the variables measured per sediment layer.

		Factors			
		*RM*		*DPD × RM*
		
Variable	Sediment layer	*F*_(2,18)_	*p*	*F*_(4,18)_	*p*
WC (%)	Surface	5.38	***0.018***	0.45	*ns*
	Deep	7.64	***0.0005***	0.13	*ns*
Sediment NO_3_^−^ (μg N g^−1^ DM)	Surface Deep	1.23 2.28	*ns* *ns*	0.36 11.62	*ns* ***0.0002***
Sediment NH_4_^+^ (μg N g^−1^ DM)	Surface Deep	4.75 4.03	***0.022*** ***0.035***	2.90 0.97	***0.05*** *ns*
Sediment DON (mg N g^−1^ DM)	Surface Deep	1.22 2.17	*ns* *ns*	2.06 0.65	*ns* *ns*
AO rates (μg NH_4_^+^-N g^−1^ DM h^−1^)	Surface Deep	0.48 39.56	*ns* ***<0.0001***	0.67 10.24	*ns* ***0.0003***
DNA (ng DNA g^−1^ DM)	Surface Deep	2.20 0.07	*ns* *ns*	0.45 0.32	*ns* *ns*
AOA (RA)	Surface Deep	3.72 1.51	***0.004*** *ns*	0.37 0.89	*ns* *ns*
Marine AOA (RA)	Surface Deep	0.45 0.61	*ns* *ns*	0.48	*ns* *ns*
Soil AOA (RA)	Surface Deep	0.98 20.88	*ns* ***<0.0001***	0.66 3.35	*ns* ***0.038***
AOB (RA)	Surface Deep	0.68 13.97	*ns* ***<0.001***	0.91 2.40	*ns* *ns*
*amoA* AOA (n° copies g^−1^ DM)	Surface Deep	0.54 3.61	*ns* ***0.048***	2.38 9.48	*ns* ***0.0003***
*amoA* AOB (n° copies g^−1^ DM)	Surface Deep	13.53	***0.0003*** ***0.05***	2.33 1.08	*ns* *ns*

*Models included the factors: dry period duration (DPD = 3, 6, and 9 weeks), rainfall magnitude (RM: 0, 4, and 21 mm) and interaction of both factors. Results from the effects of the RM and from the interaction RM × DPD are only shown. ns indicates results no significant at P-value > 0.05. WC: water content, AO: ammonia oxidation; AOA: ammonia-oxidizing archaea; AOB: ammonia-oxidizing bacteria; DM: dry mass. RA: relative abundance. Marine AOA refers to the order Nitrosopumilales while Soil AOA refers to the order Nitrososphaerales.*

### Sediment N Content

**FIGURE 2:**
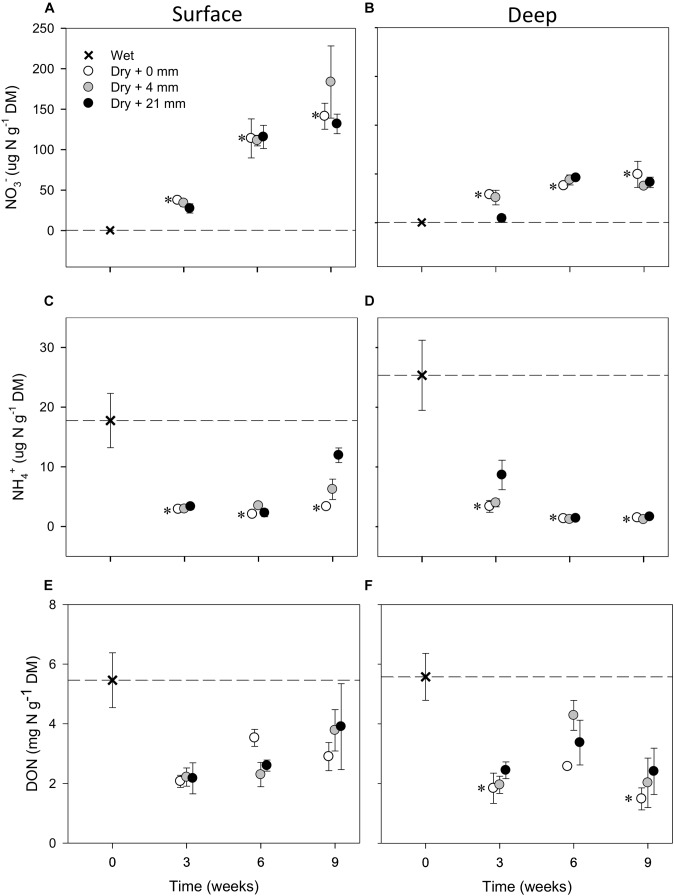
Mean (±SE, *n* = 3) concentration of NO_3_^−^, NH_4_^+^, and DON in surface **(A,C,E**, respectively**)** and deep sediments **(B,D,F**, respectively**)** during initial conditions (i.e., 0 weeks) and after the different RMs for each DPD. Asterisks (^∗^) denote significant (*P* ≤ 0.05) differences to initial wet conditions after Student’s *t*-test followed by Holm’s correction for multiple comparisons. The dashed line represents the initial conditions as a reference for a better comparison.

Compared with wet conditions (*t* = 0 weeks), NO_3_^−^ in both sediment layers significantly increased in the microcosms subjected to dry conditions (i.e., 0 mm selected; *t*-test, *P* ≤ 0.05) (Figures 2A,B). Without any rainfall, the average NO_3_^−^ in dry surface sediments increased 350 times over 6 weeks of drying (up to 113 μg N g^−1^ DM, *SD* = 42, *n* = 3) and 440 times over 9 weeks (up to 141 μg N g^−1^ DM, *SD* = 28, *n* = 3) (Figure 2A). The increase was not as high in deep sediments, where NO_3_^−^ reached 50 μg N g^−1^ DM (*SD* = 23, *n* = 3) after 9 weeks of drying without any rainfall (Figure 2B). In contrast to NO_3_^−^, NH_4_^+^ in dry sediments decreased significantly (*t*-test, *P* ≤ 0.05) after 3 weeks of drying and then remained low as drying progressed (Figures 2C,D). This NH_4_^+^ decrease was more pronounced in deep (Figure 2D) than in surface (Figure 2C) layers, mainly due to higher initial NH_4_^+^. Concentrations of DON also dropped with drying, yet variation was large and the observed differences with respect to initial wet conditions in the dry microcosms were not significant (*t*-test, *P* > 0.05) (Figures 2E,F).

The mixed effects models detected significant differences among levels of RM in deep sediments in the case of NO_3_^−^ and in both layers in the case of NH_4_^+^ (Table 1). Yet, in some instances, RM effects depended on the DPD as indicated by the significance of the interaction term (Table 1). In the case of NO_3_^−^, concentrations in deep sediments after 3 weeks were significantly lower under 21 mm of rainfall (*t*-test, *P* ≤ 0.05) (Figure 2B). In the case of NH_4_^+^, significantly higher concentrations were observed in surface sediments subjected to 4 mm and 21 mm of rainfall (*t*-test, *P* ≤ 0.05) after 6 and 9 weeks, respectively (Figure 2C).

### AO Rates

**FIGURE 3:**
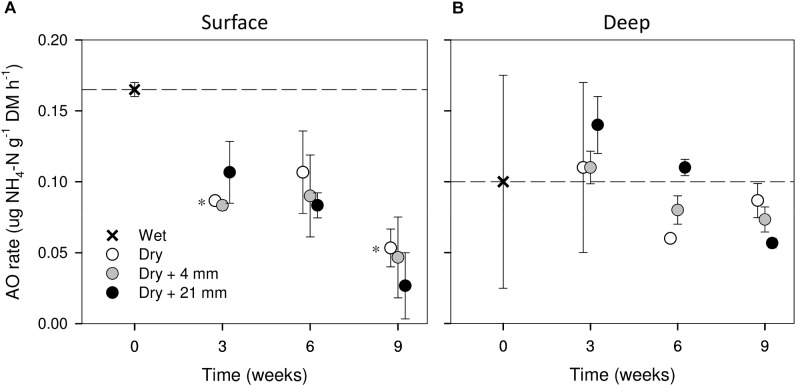
AO rates in surface **(A)** and deep **(B)** sediments during initial conditions (0 weeks) and after the different RMs for each DPD. Asterisks (^∗^) denote significant (*P* ≤ 0.05) differences to initial wet conditions after Student’s *t*-test followed by Holm’s correction for multiple comparisons. Values are means ± SE (*n* = 3). The dashed line represents the initial conditions as a reference for a better comparison.

Compared with wet conditions (*t* = 0 weeks), AO rates in surface sediments tended to decrease with drying (i.e., 0 mm selected), yet significant differences were found only after 3 and 9 weeks (*t*-test, *P* ≤ 0.05, Figure 3A). In contrast, in deep sediments AO rates remained relatively stable as drying progressed (Figure 3B). RM effects on AO rates were found only in deep sediments, albeit mediated by DPD (Table 1). In this case, the highest AO rates were observed after 6 weeks of drying under 21 mm rainfall (Figure 3B).

### Microbial Biomass, Community Dynamics, and Relative Abundance of Nitrifying Functional Groups

DNA content in sediments, as a proxy for microbial biomass, ranged from 9 to 27 and 9 to 29 μg g^−1^ DM in surface and deep layers, respectively (Supplementary Figure S2). We found no significant differences in DNA content from wet (*t* = 0 weeks) to drying conditions (i.e., 0 mm; *t*-test, *P* > 0.05) and between RM treatments (Table 1).

**FIGURE 4:**
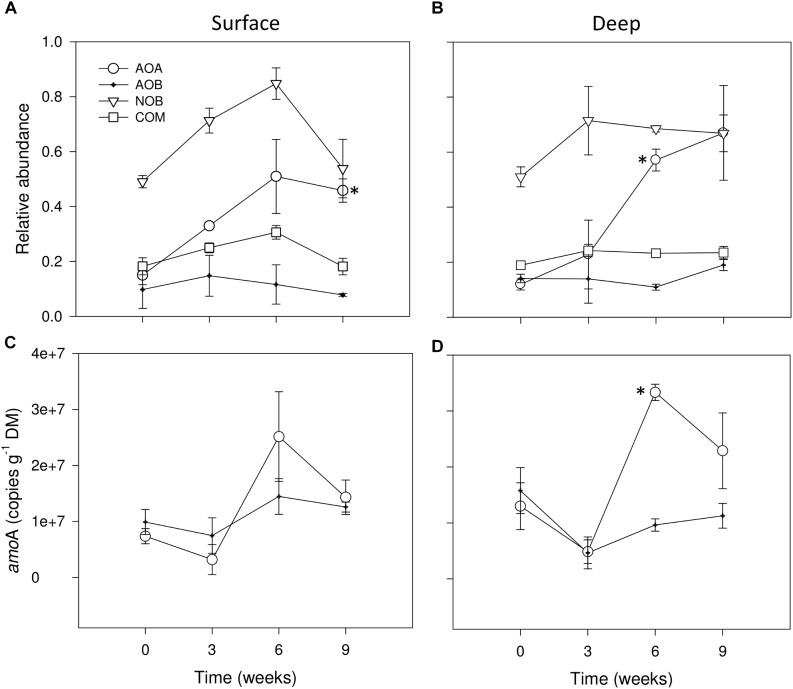
Relative abundances (%) of 16S rRNA sequence tags from AOA, AOB, NOB and COMAMMOX groups in surface **(A)** and deep sediments **(B)** during initial conditions (i.e., 0 weeks) and after the different DPDs with no rainfall (i.e., 0 mm selected). Number of copies of *amoA* genes for AOA and AOB in surface **(C)** and deep sediments **(D)** during initial conditions and after the different DPDs with no rainfall. Asterisks (^∗^) denote significant (*P* ≤ 0.05) differences to initial wet conditions after Student’s *t*-test followed by Holm’s correction for multiple comparisons. Values are means ± SE (*n* = 3).

We detected the ammonia oxidizers: AOA, AOB and we potentially identified COMAMMOX as well as NOB affiliated with the *Nitrospirales*. For a better comparative description of RA, in Figures 4A,B we grouped the results of RA of all nitrifying microbial groups, determined by analysis of 16S rRNA gene amplicon datasets concerning wet (*t* = 0 weeks) and dry conditions (i.e., 0 mm selected). We observed that none of the studied groups was highly abundant, as none of them accounted for more than 1% of the total microbiota 16S rRNA genes (Figures 4A,B). Among all nitrifying microbes, NOB were potentially the most abundant group from wet to drying conditions after 6 weeks, especially in surface sediments (Figures 4A,B), later, in both sediment layers AOA strongly increased after 9 weeks of drying (Figures 4A,B) exhibiting significant differences with wet conditions as drying progressed (*t*-test, *P* ≤ 0.05). AOB and the tentative group proposed as COMAMMOX stayed relatively constant throughout the experiment in both sediment layers (Figures 4A,B).

The mixed effects models (Table 1) showed significant differences between RM for AOA in surface sediments, for AOB in deep sediments regardless the DPD. No consistent pattern based on RM effects was found for these groups, yet higher RAs seemed to appear under 0 and 4 mm (Supplementary Figure S3).

**FIGURE 5:**
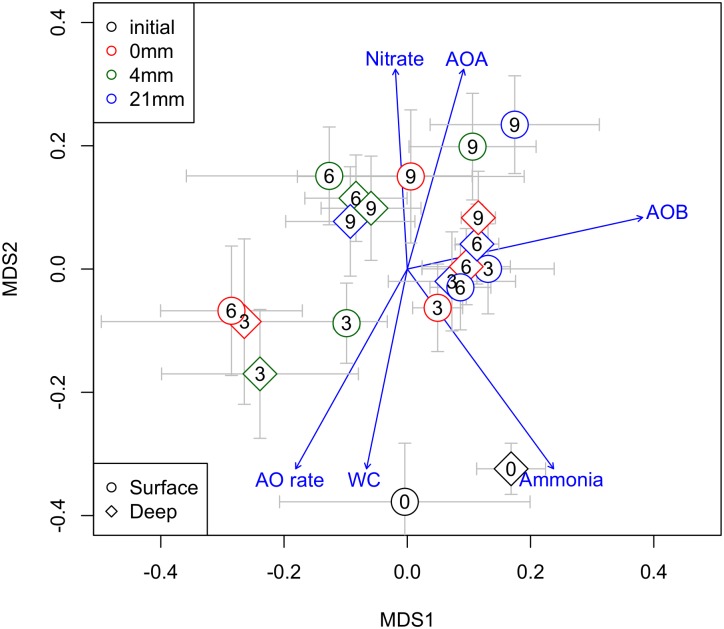
Non-metric multidimensional scaling ordination (nMDS) of all prokaryotes community showing AOA and AOB (RA) in relation to a suit of chemical and biogeochemical variables of sediments (WC%, nitrate and ammonia concentration and AO rates) potentially associated across the different DPDs. The different DPDs (*t* = 0, 3, 6, and 9 weeks) are reflected by numbers within the symbols. Different symbols shapes and colors denote different sediment layers sediments and different RMs, respectively.

The overall community dynamics of prokaryotes in the sediments, visualized by nMDS analyses (Figure 5), revealed a temporal trend from 0 to 9 weeks, presumably driven by WC. Communities at 6 and 9 weeks were associated to higher RA of AOA and sediment NO_3_^−^, while communities at 0 and 3 weeks were associated to higher AO rates, higher NH_4_^−^ and WC (Figure 5). RA of AOB varied independently of this temporal trend.

In the case of the AOA orders, the candidate order *Nitrosopumilales* (formerly known as “marine group I”) dominated over the *Nitrososphaerales* (formerly known as “soil group,” Kerou et al., 2016) during the whole experiment (Supplementary Figure S4). Despite a lack of statistical significance, the RA of *Nitrosopumilales* tended to increase with time in the dry sediments compared with wet conditions, especially in deep layers. Similarly, *Nitrososphaerales* exhibited a modest increase after 9 weeks of drying in surface sediments, while in deep sediments, no marked changes compared with wet conditions were detected in any case (Supplementary Figure S4). An effect of RM was only found for *Nitrososphaerales* and only in deep sediments after 6 weeks of drying (Table 1). In this case, the microcosms subjected to the intermediate RM of 4 mm showed the lowest abundances, while those that remained dry (i.e., 0 mm) and subjected to 21 mm had similar values (Supplementary Figure S4).

### Abundance of AOB and AOA *amoA* Genes

During wet conditions, the abundances of the functional marker genes *amoA* of AOA and AOB were similar, with average values of 7.4 10^6^ and 9.8 10^6^g^−1^ DM, respectively, in surface sediments (Figure 4C) and of 1.3 10^7^ and 1.6 10^7^, respectively, in deep sediments (Figure 4D). Overall, the number of *amoA* copies in the dry microcosms (i.e., 0 mm selected) after 3 weeks tended to drop when compared with wet conditions (*t* = 0 weeks), especially in deep sediments (Figure 4D). After 6 weeks, however, AOA *amoA* copies in both sediment layers tended to increase to values significantly higher than in wet conditions only in deep sediments (*t*-test, *P* ≤ 0.05) (Figure 4D). Later, a slight decrease of *amoA* copies for AOA was detected after 9 weeks of desiccation while for AOB it remained stable (Figures 4C,D).

As indicated by the significant interaction term (Table 1), RM had a significant effect in the case of AOA *amoA* in deep sediments only after 6 weeks, when rainfall of 0 mm (i.e., dry microcosms) generated the highest values (Supplementary Figure S3). In the case of AOB *amoA*, significant differences between RM were found in both sediment layers (Table 1). While in surface sediments, *amoA* copy numbers were higher under 0 mm for most dry periods (Supplementary Figure S3), in deep sediments, this treatment tended to support the lowest values (Supplementary Figure S3).

### N_2_O Fluxes

**FIGURE 6:**
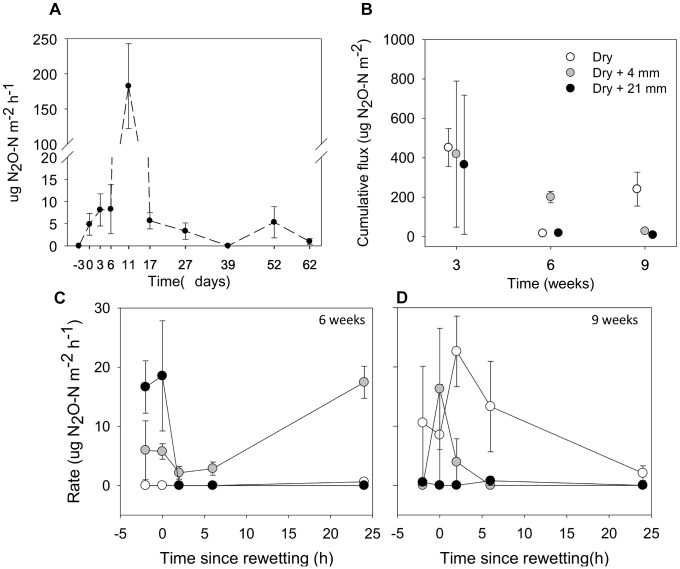
**(A)** Fluxes of N_2_O from sediments before microcosms drainage (microcosms with a surface water column; *t* = –3 days), immediately after drainage (*t* = 0 days) and several times after drainage and drying. **(B)** Accumulated fluxes of N_2_O following 24 h under the effects of variable RMs (0, 4, and 21 mm) after the different DPDs. **(C)** and **(D)** Fluxes of N_2_O before (*t* = –2 h), immediately (*t* = 0 h) and following rainfall events (*t* = 2, 6, and 24 h) after 6 and 9 weeks of drying, respectively. Values are means ± SE (*n* = 3).

Over the 9-week experiment, we observed considerable N_2_O fluxes from microcosms without rainfall (Figure 6A). From the beginning (*t* = −3 days) and over the first 2 weeks of drying, there was an increase in N_2_O fluxes, especially after 11 days of desiccation, when fluxes peaked at 182.5 μg N_2_O-N m^−2^ h^−1^. Following this peak, N_2_O fluxes decreased to low values until the end of the experiment (Figure 6A).

The cumulative flux of N_2_O estimated over 24 h tended to decrease as drying progressed in microcosms without rainfall (i.e., 0 mm selected) (Figure 6B). The mixed effects model indicated that cumulative fluxes responded to RM depending on the DPD (interaction term *F*_(4,18)_ = 8.61, *P* < 0.001). Significant differences among levels of RM were only detected after 6 and 9 weeks of drying. After 6 weeks, the highest cumulative flux was observed after the rainfall of 4 mm (mean = 200.7 μg N_2_O-N m^−2^), while after 9 weeks, it was detected under 0 mm (mean = 241.3 μg N_2_O-N m^−2^; *t*-test, *P* ≤ 0.05, Figure 6B).

In addition, after 6 and 9 weeks we thoroughly examined the development of N_2_O fluxes before and following rainfall events (Figures 6C,D). In both cases the mixed effects models showed significant differences among levels of RM on N_2_O fluxes depending on the monitoring phase (*t* = −2, 0, 2, 6, and 24 h), as indicated by the significant interaction (after 6 weeks, *F*_(2,39)_ = 9.30, *P* < 0.001; after 9 weeks, *F*_(2,39)_ = 5.32, *P* < 0.01). We must note the considerable variability of N_2_O-N fluxes that existed among treatments before rainfall (*t* = −2 h), specially marked after 6 weeks of drying (Figure 6C). After 6 weeks, the N_2_O fluxes under 0 mm rainfall remained consistently close to zero during the 24-h monitoring period. In contrast, N_2_O fluxes following the water pulses (4 and 21 mm) changed considerably over time (Figure 6C). For example, when compared with *t* = −2 h (mean = 16.65 μg N_2_O-N m^−2^ h^−1^, *n* = 3), we observed a notable reduction of the average N_2_O fluxes at *t* = 2 h after the water pulse (0 μg N_2_O-N m^−2^h^−1^) (Figure 6C). However, the rainfall of 4 mm almost doubled the average N_2_O flux at *t* = 24 h (mean = 17.45 μg N_2_O-N m^−2^h^−1^, *n* = 3) when compared with *t* = −2 h (mean = 8.95 μg N_2_O-N m^−2^h^−1^, *n* = 3) (Figure 6C). After 9 weeks, we found that when compared with 0 mm, average N_2_O fluxes following water pulses were generally low, especially under the rainfall of 21 mm (Figure 6D). Although increased N_2_O fluxes immediately after the rainfall of 4 mm (*t* = 0 h) were measured when compared with *t* = −2 h (mean from 0 to 16.28 μg N_2_O-N m^−2^h^−1^, *n* = 3), fluxes gradually fell down to zero and remained as such until the end of the monitoring period (Figure 6D).

## Discussion

Our experiment revealed considerable variations in sediment N processing and microbial communities of AOA and AOB in response to drying duration. Our prediction of increased nitrifying microbes’ abundance and activity with desiccation and differential response in surface and deep sediment layers was only partially supported by the results. As expected, we observed that the abundance of AO groups, in particular of AOA, generally increased with desiccation. Furthermore, drying progression led to an increase in sediment NO_3_^−^ and this increase was stronger in surface sediments. Contrary to our expectations, however, the AO rates did not increase with desiccation, but rather decreased (in surface sediments) or remained stable (deep sediments) over the course of the experiment.

The predicted effects of rainfall on the base of their magnitude – that is, stronger changes at higher RM - were only observed for AO rates and sediment NH_4_^+^ in surface and in deep sediments, respectively. Interestingly, this response depended on the duration of the preceding dry period, appearing only after 6 and 9 weeks in the case of AO rates and NH_4_^+^, respectively. N_2_O flux also fluctuated in association to sediment drying, and, surprisingly, its variable reaction to RM disagreed with our initial expectations. However, in terms of microbial communities, no marked changes were visible for sediment microbial communities of AOA and AOB after 24 h of our simulated rainfall.

### Abundance of Ammonia-Oxidizing Functional Groups in Response to Drying and Rainfall

All known major microbial groups involved in the AO pathway were identified over the whole experiment, including the newly described COMAMMOX group (Daims et al., 2015; van Kessel et al., 2015) that we tentatively identified and separated from NOB. We applied two independent, complementary approaches for the identification of nitrifying microbes, (1) 16S rRNA genes and (2) *amoA* gene qPCR. While the former yielded information on the taxonomy of nitrifying microbes, it only provided RA data, that are difficult, if not impossible, to relate to process measurements (Alves et al., 2013). qPCR-assays of *amoA* for two of the groups (AOA and AOB) yielded functional gene copy numbers per gram of DM, values that can be directly related to rates of AO.

For AOA and AOB, the temporal trend of abundance examined through both 16S rRNA genes and *amoA* genes provided similar information, i.e., an increasing trend of AOA abundance, while AOB abundance remained rather constant if compared with wet conditions. However, this congruence was supported only for AOA groups which related positively with *amoA* genes (Simple Linear Regression, *R*^2^ = 0.45, *P* < 0.01, *n* = 24), highlighting the difficulty of establish robust linkages between community structure and functioning features, such as the abundance of a particular functional gene (Prosser, 2012). We acknowledge the limitations of using DNA-based community profiling, as DNA is a relatively stable molecule and can remain in the environment even after the death of the cells harboring it. Therefore, temporal trends should be interpreted with caution, as there may be a significantly delayed drop in detection of dying organisms’ genes upon environmental change.

The relatively constant amount of DNA in the sediments during the whole experiment may support this fact. Likewise, the observed disagreement between AO rates measurements and microbes abundance is not simply caused by differences in the amount of extracted DNA. On the other hand, our findings enable a comparison of the RA of nitrifying groups in 16S rRNA gene amplicons with the AO process measurements.

According to our initial expectations, AOA exhibited an increasing trend until 6 weeks of drying in both surface and deep sediments. Furthermore, AOA *amoA* abundance also increased until 6 weeks of drying. Although we must be cautious when interpreting our results based on DNA data from a perspective of activity, this increasing trend in abundance, at least until 6 weeks, agrees with previous studies that suggest certain resistance to water stress of AOA (Merbt et al., 2016).

On the contrary, the observed drop in the RA of AOA and *amoA* genes after 9 weeks could indicate sensitivity of AOA to extreme desiccation frequently associated to long dry periods. In agreement with this finding, Thion and Prosser (2014) reported negative impacts of dry conditions on AOA *amoA* gene abundance in grassland soils.

Interestingly, within the AOA groups, we found that the RA of the “marine order” *Nitrosopumilales* tended to fall rather than increase in surface sediments after 9 weeks of drying while soil AOA clades kept increasing. This result points to the notion of dry, air-exposed conditions as the possible selecting force for *Nitrososphaerales* (the former soil group) over *Nitrosopumilales* (the former marine group I) (Tourna et al., 2011; Stieglmeier et al., 2014). In fact, we found that the RA of *Nitrososphaerales* showed a negative relationship with WC% in surface sediments (Simple Linear Regression, *R*^2^ = −0.42, *P* < 0.001, *n* = 29). Results in this respect also support the idea of the replacement of species typical of aquatic habitats by those more frequently dominating terrestrial systems, as has been previously suggested in intermittent rivers during non-flow periods (Timoner et al., 2014; Sabater et al., 2016).

In most cases, rainfall events (either modest or intense) had no major effects on the RA of nitrifying groups and number of *amoA* copies. A plausible explanation for this lack of marked differences can lie in the sampling time we use to collect the sediments after rainfall (24 h following rainfalls) which could not be long enough to visualize changes driven by RMs, at least in terms of DNA. In water-limited soils, increased microbial activity in terms of biogeochemical rates has been reported in response to water pulses within timeframes of minutes (Schwinning and Sala, 2004). However, changes in activity may not be translated into changes in the abundance within the same time frame.

In some instances we found decreased RA in response to rainfall. After the longest dry period (i.e., 9 weeks) the RA of AOB (in deep layer) decreased in response to rainfall (both after 4 and 21 mm). Studies from soils have shown how sudden rewetting events after drying can exert drastic shifts in matrix potential, inducing cellular lysis and altering microbial community composition (Kieft et al., 1987; Fierer et al., 2003; Gordon et al., 2008), a situation that could be behind these findings. However, our DNA-based approaches do not allow assessments of microbial activity due to potential retention of “dead” DNA in the environment. The constant sediment DNA content over time in our experiment, may reflect both living biomass and dead material. Our inconclusive findings in this respect highlight the importance of including molecular analyses dealing with functional aspects (e.g., transcriptomics and activity measurements) to achieve deeper information whether and when changes in the humidity of sediments impact on microbial communities in streambeds.

Finding links of ammonia-oxidizing microbes abundance and functional genes with ecosystem process measurements is tough (Prosser, 2012). In our experiment, we did not find any direct relationship between the RAs of these groups and AO rates. Also, AO rates were not correlated with the quantitative data, AOA *amoA* and AOB *amoA* copy numbers. Previous studies in marine habitats and streams have also reported such lack of correspondence despite observing high AOA *amoA* abundances (Santoro et al., 2010; Merbt et al., 2016) as in our case. High abundance of AOA does not mean AOA are driving AO, since the cell-specific AO rates are generally higher for AOB than for most AOA (Alves et al., 2013). Truly understanding such linkages is additionally complicated by new discoveries about physiological versatility in some groups, such as the NOB and COMAMMOX (Daims et al., 2016; Kits et al., 2017), preventing the clear assignment of microbial taxa to functional guilds. Nevertheless, when data were pooled across both sediment layers, we found evidence for synchrony between the variation of marine AOA (*Nitrosopumilales*) and sediment NH_4_^+^ (Simple Linear Regression, *R*^2^ = −0.43, *P* < 0.001, *n* = 56) which suggests that this AOA group could be the driving force behind AO variation in our experiment and as such could contribute to dropping NH_4_^+^.

### AO and N Pools in Response to Drying and Rainfall

AO rates decreased strongly in surface sediment but remained stable in the deep sediment layer. Simultaneously, NO_3_^−^ increased steadily and stronger in surface than in deep sediment, while NH_4_^+^ dropped markedly within the first 3 weeks and then remained low in both sediment layers. In soils, nitrification activity has been reported to be low in water-saturated conditions (due to reduced oxygen diffusion) as well as at very low moisture (Rennenberg et al., 2009; Thion and Prosser, 2014). In our microcosms, suboptimum conditions for nitrification were likely associated to low oxygen levels observed at initial wet conditions (*t* = 0 weeks). Far from observing increased AO rates from wet to dry conditions due to enhanced oxygen diffusion, AO rates appeared decreased after the study dry periods, especially in surface sediments. Certain water stress as a result of loss of sediment moisture over time could be a plausible explanation for continuously low AO rates. In line with these findings, Austin and Strauss (2011) also found decreased AO rates in an experimental intermittent stream after 28 days of desiccation, likely due to water stress. However, deep layers, despite not allowing large oxygen diffusion as in surface, are more protected to desiccation and can retain more water to avoid a strong reduction in the biogeochemical activity. Contrary to our findings, Merbt et al. (2016) detected increased AO rates with drying of an intermittent stream. However, their “drying” gradient was not established along a temporal sequence in the same habitat, but across different habitats with potentially different environmental conditions controlling AO rates, such as NH_4_^+^ availability.

The notion of water limitation for AO activity in our experiment was also suggested during the water pulses, when the rainfall event greater in magnitude (i.e., 21 mm) triggered higher AO rates in deep layers. Such spikes indicate that the impact of drying on N processing can be alleviated after a certain time of desiccation by an adequate increase of sediment moisture. In fact, in our experiment, WC% was the only variable significantly related with AO rates (Simple Linear Regression, *R*^2^ = 0.42, *P* < 0.001, *n* = 56, data pooled across sediment layers). As in arid and semiarid soils (Austin et al., 2004; Schwinning et al., 2004), in our microcosms the duration of the previous dry period determined the response to a water pulse. Interestingly, the triggered AO activity in deep sediments in response to rainfall was observed after 6 weeks of drying, and despite not significant, also a modest increase after 3 weeks was detected. Thus, short and intermediate dry phase duration could be optimally supporting the positive effect of a following water pulse on AO. On the contrary, after 9 weeks, physiological constraints caused by strong desiccation (e.g., cell dormancy) may hinder the response of microorganisms to water pulses. Such physiological constraints could also explain the lack of a significant response of AO to even the most intense water pulses in the more water-stressed surface sediments.

Despite the general reduction in AO rates observed in our experiment, we cannot rule out an unobserved increase in AO rates in surface sediments occurring at very early desiccation before our first sampling, as has been reported by others. In an experimental streambed, Gómez et al. (2012) observed high accumulation of NO_3_^−^ during the first 10 days of drying followed by a notable reduction likely due to microbial assimilation. Unobserved increased AO rates during the early phase of the experiment may have contributed to the large drop of NH_4_^+^ observed after 3 weeks onward, which in turn may have further decreased AO rates due to N limitation later in the experiment. NH_4_^+^ is a master driver of N cycling processes and is naturally crucial for AO (Verhamme et al., 2011). Diminishing NH_4_^+^ concentration, either due to competition for NH_4_^+^ with heterotrophs (Strauss et al., 2004) or due to reduced ammonification could also contribute to the observed general decrease in AO rates. The general drop in DON with drying with minimal differences among RMs points to N mineralization at least during the first 3 weeks of desiccation. Later, however, constrained microbial activity by water stress likely restrained ammonification as a source of inorganic N for AO as our experiment progressed. The sudden increase in NH_4_^+^ following rainfall in surface sediments after 9 weeks of drying could be due to osmotic shock and release of cellular NH_4_^+^ (Halverson et al., 2000). Interestingly, this NH_4_^+^ “flash” did not translate into an increased AO rate likely because water limitation was more important for AO than substrate availability after 9 weeks.

Last, a lack of an external source of NH_4_^+^ in our microcosms could also contribute to explain the low reactivity of AO rates to the rainfall treatments we found in many cases. Rainwater usually contains NH_4_^+^ and NO_3_^−^. However, in our experiment we avoided enriching our artificial rainwater with inorganic N to prevent confounding effects with riverbed humidity as both factors can limit AO rates. Therefore, we must be cautious when extrapolating our lab results with respect to RMs to the real environment since we could observe a different effect. We must note that in the natural environment the NH_4_^+^ inputs that frequently accompany precipitation events may trigger important “hot moments” for nitrification by alleviating not only water stress but also a potential NH_4_^+^ limitation.

Interestingly, we must highlight that despite the observed low AO rates, NO_3_^−^ continued to increase steadily until the end of the experiment, suggesting sustained nitrification activity. The relatively lower increase in deep compared to surface sediments after 6–9 weeks of drying (from 0.23 to 50 μg N g^−1^ DM) may be at least partially explained by a more likely relevant role for denitrification and NO_3_^−^ removal in deep sediments, since denitrification is substantially reduced in exposed, dry sediments (Austin and Strauss, 2011; Gómez et al., 2012). Despite this vertical stratification, with respect to NO_3_^−^, our results generally support previous studies that demonstrate that temporal progression of drying in stream sediments drives a considerable increase of mineral N pointing to dry riverbeds as potential sources of NO_3_^−^ upon flow recovery (Gómez et al., 2012; Arce et al., 2014; Merbt et al., 2016).

Finally, we must keep in mind that in our microcosms, we simulated rainfall events that did not create overlaying water and allowed relatively good oxygen diffusion to sediments. Occasionally, rewetting after considerable rainfalls drives sediment patches with limited oxygen diffusion and generates temporary anoxic pools that may result in unsuitable habitats for aerobic ammonia oxidizers but sufficient for denitrifying microbes, which ultimately alters the spatial distribution of N-pools during non-flow periods (Arce et al., 2015). Consequently, transitory conditions of high NH_4_^+^ relative to NO_3_^−^ may occur in dry riverbed sediments in response to changes in the dominant microbial community associated to redox conditions after a rewetting event (Gómez et al., 2017; von Schiller et al., 2017).

### Variation of N_2_O Fluxes Associated to Drying and Rainfall

As initially predicted, results from our experiment confirm that temporal progression of drying causes notable fluctuations of N_2_O fluxes from streambeds. We found a gradual increase of N_2_O fluxes during initial drying, with a peak average value after 11 days (182.5 μg N_2_O-N m^−2^ h^−1^). Afterward, N_2_O fluxes steeply decreased to concentrations ranging between 0 and 5.6 μg N_2_O-N m^−2^ h^−1^. The temporal behavior in N_2_O fluxes can be attributed to a combination of physical and microbial changes coupled to sediment water and oxygen fluctuations. While overlaying water slowed down N_2_O diffusion to the headspace of the microcosms at *t* = −3 days, increased gas diffusion may have favored N_2_O evasion as detected from *t* = 0 to 11 days. This pulse in gas emissions during reduction of the water table was also observed in inundated wetlands (Freeman et al., 1993; Fenner and Freeman, 2011) temporary ponds (Catalán et al., 2014) and streambed sediments (von Schiller et al., 2014; Gómez-Gener et al., 2015, 2016).

Denitrification and nitrification are the main sources of N_2_O, representing around 70% of the annual N_2_O flux from ecosystems to the atmosphere (Stevens et al., 1997; Morse and Bernhardt, 2013). In our microcosms experiment, we cannot distinguish which source was behind N_2_O fluxes and how it changed over time. However, differences in the degree of contribution on the base of their redox requirements as drying progressed may be anticipated (Bateman and Baggs, 2005; Congreves et al., 2018). For example, the first 11 days of drying, when the maximum flux was detected, WC% varied from 28 to 22% providing wet-saturated conditions and anaerobiosis, which could have promoted N_2_O emissions primarily via denitrification (Burgin and Groffman, 2012; Congreves et al., 2018). Using a ^15^N tracer approach Morse and Bernhardt (2013) found nitrification to contribute significantly, even higher than denitrification, to N_2_O yields from abundant NH_4_^+^ in organic sediments after drainage. Thus, N_2_O derived from nitrification could take place in our microcosms as DO increased. In fact, such a peak of N_2_O observed after 11 days of drying prompts us to suspect that we probably missed a “hot moment” of increased AO in the sediments before 3 weeks (i.e., the first sampling time). As desiccation advances, the flux of N_2_O via microbial pathways is expected to be low due to water constraints and associated limitations (e.g., low substrate diffusivity, Congreves et al., 2018).

Rainfall events did not have the expected positive effect based on their magnitude. However, the implications of rainfall on N_2_O emissions we detected were modulated by the duration of the precedent dry period. Significant differences of cumulative fluxes were detected after 6 and 9 weeks of drying, likely when induced moisture changes by rainfalls were more evident. Surprisingly, far from observing an increasing reaction, rewetting seemed to negatively impact emissions of N_2_O as its magnitude increased. In fact, after 6 weeks of drying, intense rainfall (i.e., 21 mm) produced a steep drop in the N_2_O flux at *t* = 2.5 h, and, unexpectedly, after 9 weeks the highest fluxes were detected in the dry sediments (i.e., 0 mm). Only in the case of the modest rainfall (i.e., 4 mm) after 6 and 9 weeks of drying, we found an immediate positive reaction (*t* = 0 h) following that pulse or a full day later (*t* = 24 h). Thus, our results partly disagree with studies from soils, which generally report an increase of N_2_O emissions following water addition (as reviewed in Kim et al., 2012). They also partially contradict results by Gallo et al. (2014), who reported increased N_2_O emissions after water pulses due to physical release and microbial activation in ephemeral streams in South Eeast United States. As in our experiment, Muhr et al. (2008) did not detect any increase of N_2_O fluxes in dry forested soils after rewetting events between 8 and 50 mm. The authors attributed their observations to limited N mineralization providing inorganic-N substrate and low microbial activity in the precedent dry conditions. Anaerobiosis and increased C and N substrated are frequently associated to large microbial N_2_O fluxes upon rewetting (as reviewed in Congreves et al., 2018). In our microcosms, intense rainfall events could support microbial N_2_O emissions in dry sediments but could have also exerted a physical trapping that hinder gas exchange and displacement, thus resulting in an apparent absence of gas fluxes during the 24 h of monitoring period.

Although we obtained data on abundance and biogeochemical activity of nitrifying microbial communities, attributing N_2_O emissions to their relative importance is complex. AOB and AOA can produce N_2_O either enzymatically via nitrifier denitrification or abiotically via reduction of NO (Kozlowski et al., 2016). Moreover, although there is no empirical evidence that COMAMMOX produce N_2_O, it has been suggested that they probably do, because their AO pathway is similar to the classic AO pathway (Kuypers, 2015). Based on the predominance of AOA over AOB in oxic environments (Nicol et al., 2008; Schauss et al., 2009; Zhang et al., 2009), AOA have been proposed as the major source of N_2_O in natural ecosystems (Santoro et al., 2011; Jung et al., 2014). Unfortunately, our headspace measurements alone make resultant N_2_O yields notoriously difficult to elucidate regarding the relative importance of sources and microbial groups.

## Conclusion

We detect and quantify the two main groups of microbes driving the first step of nitrification (AOA and AOB) in riverbed sediments under drying-rewetting conditions and we tentatively identify NOB and the newly described COMAMMOX. In addition, we provide information on their biogeochemical functioning. By combining sequencing of 16S rRNA and qPCR of *amoA* genes, we show that the abundance of nitrifying microbes and functional genes are not strongly constrained by the desiccation stress. This lack of a negative effect of drying was especially marked for AOA, *amoA* AOA before 6 weeks of desiccation. However, after 9 weeks of drying, especially in surface sediments, we detected a drop in the abundance of *amoA* AOA genes. Although these results must be considered within the context that they do not strictly represent functioning, collectively they indicate some sensitivity of AOA under extreme desiccation after longer dry periods. In parallel, AO rates tended to fall during the experiment, especially in surface sediments. We suggest that the variability observed in AO rates was more directly dependent on physical controls, primarily sediment WC% and indirectly NH_4_^+^ availability, than on biotic factors related to the relative variations in abundance of the different AO groups. Nevertheless, continued AO activity resulted in a notable accumulation of NO_3_^−^ and a drop in NH_4_^+^ as drying continued especially in surface layers. Our experimental results support the notion that surface riverbed sediments, rich in NO_3_^−^, may act as a relevant natural source of this mobile form of N to water column upon flow resumption. Furthermore, our experiment demonstrates that drying can drive important fluctuations in the flux of N_2_O to atmosphere in riverbed sediments. While N_2_O emissions, through either physical evasion or microbial activity, can be favored at early drying, N_2_O emissions after longer dry periods tend to drop. We found that even under very dry conditions, rainfall events could foster N_2_O emissions, depending on DPD and RM. Together, our results show that dry riverbeds can be active biogeochemical processors, and suggest that dry-phase processing of N, including that under sporadic rainfall events, could significantly contribute to N budgets (dissolved and gaseous) along intermittent fluvial networks.

## Author Contributions

MA, DS, and GS conceived and designed the experiments and wrote the manuscript. MA and HJ made the field and laboratory work for chemical and biogeochemical analysis. HJ and GS performed the statistical analyses. MA made the DNA extractions. MB, TU, CH, and RA made the qPCR for *amoA* genes and analyzed 16sRNA sequences. MB and TU contributed to write the manuscript.

## Conflict of Interest Statement

The authors declare that the research was conducted in the absence of any commercial or financial relationships that could be construed as a potential conflict of interest.

## Footnotes

1 http://www.r-project.org

## Abbreviations

*amo*A: ammonia monooxygenase subunit A
AO: ammonia oxidation
AOA: ammonia-oxidizing archaea
AOB: ammonia-oxidizing bacteria
COMAMMOX: complete ammonia oxidation to nitrate
DM: dry mass
DO: dissolved oxygen
DPD: dry period duration
NOB: nitrite-oxidizing bacteria
RM: rainfall magnitude
WC: water content.
