# Supplementary material for: Drying and Rainfall Shape the Structure and Functioning of Nitrifying Microbial Communities in Riverbed Sediments
Source: Front Microbiol. 2018 Nov 16;9:2794. doi: 10.3389/fmicb.2018.02794 (PMC6250940; doi:10.3389/fmicb.2018.02794)
Supplement: Supplementary file 1 [file Data_Sheet_1.docx]

Supplementary Material

**Drying and rainfall shape the structure and functioning of nitrifying microbial communities in riverbed sediments**

Maria Isabel Arce*, Daniel von Schiller, Mia M. Bengtsson, Christian Hinze, Hoseung Jung, Tim Urich and Gabriel Singer

*** Correspondence:** Maria Isabel Arce, Leibniz-Institute for Freshwater Ecology and Inland Fisheries, Müggelseedamm 301, 12587 Berlin, Germany. Tlf: +49 30 64181602. Email: marisarce@cebas.csic.es

## Supplementary Figures

**Supplementary Figure 1** Schematic description of the microcosms set up and experimental design

**Supplementary figure 2**. DNA content (ng g^-1^ DM) in surface (**A**) and deep (**B**) sediments during initial conditions (0 weeks) and after the different rainfall magnitudes for each dry period duration. Values are means±SE (n=3). The dashed line represents the initial conditions as a reference for a better comparison.

**Supplementary figure 3**. Relative abundances (%) of 16S rRNA sequence tags from AOA, AOB in surface **(A**, **C)** and deep sediments **(B**, **D)** during initial conditions (0 weeks) and after the different rainfall magnitudes for each dry period duration. Number of copies of *amoA* genes for AOA and AOB in surface **(E, G)** and deep sediments **(F, H**) during initial conditions (0 weeks) and after the different rainfall magnitudes for each dry period duration. Values are means±SE (n=3). The dashed line represents the initial conditions as a reference for a better comparison.

**Supplementary figure 4**. Relative abundances (%) of 16S rRNA sequence tags for soil AOA and marine AOA in surface **(A**, **C**, respectively**)** and deep sediments **(B**, **D**, respectively**)** during initial conditions (0 weeks) and after the different rainfall magnitudes for each dry period duration. Values are means±SE (n=3). The dashed line represents the initial conditions as a reference for a better comparison.
